# Supplementary material for: Modelling of amino acid turnover in the horse during training and racing: A basis for developing a novel supplementation strategy
Source: PLoS One. 2020 Jan 3;15(1):e0226988. doi: 10.1371/journal.pone.0226988 (PMC6941815; doi:10.1371/journal.pone.0226988)
Supplement: S1 Table — The compositions for faeces and skin in sweat were taken as averages of the body proteins presented in Table 1. The urine proportions were taken from the data in Table 2. The sweat fluid abundances were derived from the data published for sweat in Standardbred horses (Dunstan et al., 2015). The sweat latherin composition was taken from the relative abundances published previously (Beeley et al., 1986). Each contribution was applied according to published rates of excretion as indicated for each component. (PDF) [file pone.0226988.s001.pdf]

S1 Table. The percentage relative abundances for the amino acids used in determining excretion rates/day.

The compositions for faeces and skin in sweat were taken as averages of the body proteins presented in Table 1. The urine proportions were taken from the data in Table 2. The sweat fluid abundances were derived from the data published for sweat in Standardbred horses (Dunstan et al., 2015). The sweat latherin composition was taken from the relative abundances published previously (Beeley et al., 1986). Each contribution was applied according to published rates of excretion as indicated for each component.

Excretion route	His	Ser	Gly	Lys	Asp	Glx	Orn	Leu	Ile	Val	Thr	Met	Tyr	Phe	Pro	Ala
**Urine** *202mg/Kg BW/day*	1.9%	8.0%	8.8%	3.8%	0.1%	28.1%	3.4%	0.3%	0.0%	0.7%	2.4%	0.8%	0.7%	0.7%	0.1%	1.8%
**Faeces** *41mg/Kg BW/day*	3.2%	4.5%	10.6%	8.0%	8.6%	11.7%		8.7%	3.6%	5.3%	4.8%	1.9%	2.9%	4.0%	8.1%	8.2%
**Sweat fluid** *5mg/Kg BW/day*	6.4%	24.6%	12.7%	4.7%	4.8%	5.5%	1.2%	4.0%	0.0%	4.3%	0.0%	0.2%	2.7%	4.1%	3.5%	15.2%
129mg **Sweat latherin** *78mg/Kg BW/day*	0.5%	6.0%	6.3%	1.9%	14.4%	11.9%		24.5%	7.2%	5.5%	3.9%	0.0%	1.4%	1.8%	5.0%	4.5%
**Sweat Skin** *46mg/Kg BW/day*	3.2%	4.5%	10.6%	8.0%	8.6%	11.7%		8.7%	3.6%	5.3%	4.8%	1.9%	2.9%	4.0%	8.1%	8.2%
$\%AA_{Horse\ exc}$	**2.0%**	**7.1%**	**8.7%**	**4.4%**	**5.1%**	**20.6%**	**1.8%**	**2.4%**	**2.8%**	**3.2%**	**3.2%**	**0.9%**	**1.4%**	**1.8%**	**3.1%**	**4.0%**
