# Supplementary material for: Modelling of amino acid turnover in the horse during training and racing: A basis for developing a novel supplementation strategy
Source: PLoS One. 2020 Jan 3;15(1):e0226988. doi: 10.1371/journal.pone.0226988 (PMC6941815; doi:10.1371/journal.pone.0226988)
Supplement: S2 Table — (PDF) [file pone.0226988.s002.pdf]

S2 Table. The amino acid contents for each feed were adjusted to g / g food source and then multiplied based on a daily rations to derive a tailored mix.

		His	Ser	Gly	Lys	Asp	Glx	Leu	Ile	Val	Thr	Met	Tyr	Phe	Pro	Ala
	legume	0.022	0.051	0.054	0.060	0.122	0.112	0.086	0.048	0.058	0.051	0.015	0.036	0.053	0.114	0.066
	Corn	0.027	0.049	0.047	0.030	0.065	0.210	0.110	0.039	0.047	0.026	0.014	0.040	0.049	0.089	0.078
	Oats	0.023	0.043	0.050	0.043	0.091	0.246	0.076	0.040	0.051	0.034	0.026	0.032	0.054	0.048	0.051
	Lucerne	0.031	0.052	0.046	0.060	0.191	0.094	0.075	0.042	0.060	0.051	0.008	0.046	0.045	0.090	0.060
	Barley		0.064	0.065	0.026	0.060	0.230	0.078	0.037	0.066	0.043	0.014	0.021	0.048	0.159	0.064
	Wheat	0.020	0.054	0.064	0.020	0.041	0.349		0.043	0.052	0.030	0.017	0.019	0.047	0.154	0.046
Ration per day																
2	legume	0.044	0.102	0.108	0.12	0.244	0.224	0.172	0.096	0.116	0.102	0.03	0.072	0.106	0.228	0.132
1	Corn	0.027	0.049	0.047	0.030	0.065	0.210	0.110	0.039	0.047	0.026	0.014	0.040	0.049	0.089	0.078
2	Oats	0.046	0.086	0.1	0.086	0.182	0.492	0.152	0.08	0.102	0.068	0.052	0.064	0.108	0.096	0.102
2	Lucerne	0.062	0.098	0.094	0.060	0.130	0.420	0.220	0.078	0.094	0.052	0.028	0.080	0.098	0.178	0.156
2	Barley	0	0.128	0.13	0.052	0.12	0.46	0.156	0.074	0.132	0.086	0.028	0.042	0.096	0.318	0.128
1	Wheat	0.020	0.054	0.063	0.020	0.040	0.348	0	0.043	0.052	0.030	0.017	0.019	0.047	0.154	0.046
	Sum	0.199	0.517	0.543	0.368	0.782	2.155	0.810	0.410	0.543	0.364	0.169	0.317	0.504	1.063	0.642
	% for rationed mix	2.04%	5.29%	5.55%	3.77%	8.00%	22.05%	8.29%	4.20%	5.56%	3.73%	1.73%	3.24%	5.16%	10.88%	6.57%
1	legume	0.022	0.051	0.054	0.060	0.122	0.112	0.086	0.048	0.058	0.051	0.015	0.036	0.053	0.114	0.066
1	Corn	0.027	0.049	0.047	0.030	0.065	0.210	0.110	0.039	0.047	0.026	0.014	0.040	0.049	0.089	0.078
1	Oats	0.023	0.043	0.050	0.043	0.091	0.246	0.076	0.040	0.051	0.034	0.026	0.032	0.054	0.048	0.051
2	Lucerne	0.062	0.104	0.092	0.120	0.382	0.188	0.150	0.084	0.120	0.102	0.016	0.092	0.090	0.180	0.120
2	Barley	0.000	0.128	0.130	0.052	0.120	0.460	0.156	0.074	0.132	0.086	0.028	0.042	0.096	0.318	0.128
2	Wheat	0.041	0.108	0.127	0.040	0.081	0.697	0.000	0.087	0.104	0.060	0.035	0.038	0.094	0.309	0.093
	Sum	0.175	0.483	0.500	0.345	0.861	1.913	0.578	0.372	0.512	0.359	0.134	0.280	0.436	1.058	0.536
	%	1.95%	5.44%	5.53%	4.12%	10.40%	19.82%	7.32%	4.16%	5.83%	4.17%	1.47%	3.31%	4.93%	11.45%	6.20%
