# Supplementary material for: Modelling of amino acid turnover in the horse during training and racing: A basis for developing a novel supplementation strategy
Source: PLoS One. 2020 Jan 3;15(1):e0226988. doi: 10.1371/journal.pone.0226988 (PMC6941815; doi:10.1371/journal.pone.0226988)
Supplement: S3 Table — Comparisons of the excretion losses and nitrogen balances for the amino acids using (a) the average amino acids of the six food sources reported in Table 2 and (b) using the rationed mix described in Table A2. (PDF) [file pone.0226988.s003.pdf]

S3 Table. Comparisons of the excretion losses and nitrogen balances for the amino acids using (a) the average amino acids of the six food sources reported in table 2 and (b) using the rationed mix described in Table A2.

	Results using average composition of amino acids in the six sources of food presented in Table 2.	Results using average composition of amino acids from the rationed mix containing legume: corn: oats: lucerne: barley: wheat 2:1:2:2:2:1	Results using average composition of amino acids from the rationed mix containing legume: corn: oats: lucerne: barley: wheat 1:1:1:2:2:2
Amino acid (AA)	*Nitrogen balance using measured rates of excretion*	*Nitrogen balance using measured rates of excretion*	*Nitrogen balance using measured rates of excretion*
histidine	2.5	2.2	2.1
serine	-3.0	-3.0	-2.8
glycine	1.8	1.9	2.0
ornithine	-3.4	-3.4	-3.4
lysine	5.4	5.3	5.4
threonine	3.4	3.2	3.5
valine	5.5	5.4	5.6
leucine	17.4	17.3	15.7
isoleucine	2.9	2.9	2.9
Glutamine/glutamic acid (Glx)	-5.7	-4.6	-5.0
aspartic acid	12.4	11.1	12.5
methionine	2.2	2.3	2.1
tyrosine	4.2	4.2	4.2
phenylalanine	6.7	6.9	6.7
proline	15.6	15.6	16.5
alanine	9.4	9.8	9.3
	+77.2g	+77.2g	+77.2g
