# Supplementary material for: Modelling of amino acid turnover in the horse during training and racing: A basis for developing a novel supplementation strategy
Source: PLoS One. 2020 Jan 3;15(1):e0226988. doi: 10.1371/journal.pone.0226988 (PMC6941815; doi:10.1371/journal.pone.0226988)
Supplement: S4 Table — (PDF) [file pone.0226988.s004.pdf]

S4 Table. The adjusted nitrogen balances generated from the model under recommended feeding regime of 1.72g/Kg BW/day compared with double this rate at 3.0g/Kg BW/day for a 500Kg horse where the animal was undertaking a heavy work load and the protein turnover rate was 4 g/kg/day.

Amino acid	Adjusted nitrogen balance<br><br>Protein intake 1.7g/Kg BW/day<br><br>g	Adjusted nitrogen balance<br><br>Protein intake 3.0g/Kg BW/day<br><br>g
Histidine	2.5	4.1
Serine	-3.0	0.3
Glycine	1.8	5.3
Ornithine	-3.4	-3.4
Lysine	5.5	8.0
Threonine	3.4	5.9
Valine	5.5	9.0
Leucine	17.4	22.9
Isoleucine	2.9	5.6
Glx	-5.7	7.5
Aspartic acid	12.4	19.7
Methionine	2.2	3.2
Tyrosine	4.2	6.5
Phenylalanine	6.7	10.1
Proline	15.6	23.3
Alanine	9.4	13.8
