# Supplementary material for: Modelling of amino acid turnover in the horse during training and racing: A basis for developing a novel supplementation strategy
Source: PLoS One. 2020 Jan 3;15(1):e0226988. doi: 10.1371/journal.pone.0226988 (PMC6941815; doi:10.1371/journal.pone.0226988)
Supplement: S1 Example calculation — (PDF) [file pone.0226988.s005.pdf]

S1 Example calculation. Histidine in Table 5:

An example of the calculations for Table 5 has been provided for histidine with reference to the data presented in Table 3.

(A) Amino acids from protein intake = $\%AA_{Food}$ x 860g/day

On the basis that 860g protein were ingested per day

The average composition in ingested proteins, generated from appraisal of food sources for histidine in Table 2, was 2.5% (actually 2.466%).

Thus A = 0.02466 x 860 = 21.2 g /day

(B) Amino acids from endogenous protein turnover = $\%AA_{body}$ x 2,000g/day

The average composition in body proteins generated from appraisal of key proteins in the body for histidine in Table 2 was 3.2% (actually 3.238%).

Thus B = 0.03238 x 2,000 = 64.76 g /day (rounded to 64.8 g/day)

(C) Usage of amino acids for protein synthesis from ingested proteins was based on a published evaluation by *(Tessari, 2006)* and was set at 75% of intake. Usage of amino acids from protein turnover was based on a published evaluation by *(Tessari, 2006)* at 2/3 and was set at 66.67 % of turnover (rounded to 67%).

The amount of histidine used in protein synthesis from ingested proteins was thus 0.75 x 21.2 = 15.9 g

The amount of histidine used in protein synthesis from endogenous protein turnover was thus 0.6667 x 64.76 = 43.17g

Combined, these represent a usage of – 59.07g (rounded to 59.1 g/day)

(D) Usage of amino acids for oxidation from ingested proteins was based on a published evaluation by *(Tessari, 2006)* and was set at 15% of intake. Usage of amino acids for oxidation from protein turnover was based on a published evaluation by *(Tessari, 2006)* and was set at 27% of turnover.

The amount of histidine used in protein synthesis from ingested proteins was thus 0.15 x 21.2 = 3.18 g

The amount of histidine used in protein synthesis from endogenous protein turnover was thus 0.27 x 64.76 = 17.485 g

Combined, these represent a usage of – 20.665 g (rounded to 20.7 g/day)

(E) Excretion of amino acids (obligatory nitrogen losses, ONL) was based on published rates as outlined in Table 2 and the Materials and Methods at 372mg/Kg BW/day and the proportions of amino acids were used in the same proportions as contributions from ingested protein and that were derived from endogenous protein turnover.

The proportion of protein derived from protein ingestion was 860/2,860= 0.3007.

The proportion of protein derived from endogenous protein turnover was 2,000/2,860 = 0.6993.

The body mass was 500Kg, the obligatory nitrogen loss (ONL) was 372mg/Kg BW/day and thus the excretion losses were 500 x 0.372 = 186 g protein equivalents/day.

The average histidine composition in ingested proteins, generated from appraisal of food sources in Table 2, was 2.5%.

Thus the proportion of histidine excreted per day from the ingested protein was 0.3007 x 0.02465 x 186 = 1.3787g

The average composition in body proteins generated from appraisal of key proteins in the body for histidine in Table 2 was 3.2%.

Thus the proportion of histidine excreted per day from the endogenous protein turnover was 0.6997 x 0.03238 x 186 = 4.21g

Combined, these represent a usage of – 5.59g (rounded to -5.6g in Table 5)

F) The nitrogen balance assuming that the amino acids are lost in the same proportions as they are taken in was calculated by

Nitrogen balance g/Kg BW/day=Nitrogen intake g/Kg BW/day – Nitrogen losses g/Kg BW/day

This was calculated by A + B + C + D + E = 0.625g histidine /day for a 500Kg individual (rounded to 0.6g in Table 5)

(G) The excretion losses in urine, faeces and sweat. Excretion of amino acids in the horses was based on the obligatory nitrogen losses published by *(Tessari, 2006)* at 372mg/Kg BW/day but the proportions assigned to the losses in urine and sweat were derived from average published values. This reflected that certain amino acids were lost in urine and faeces at disproportionately faster rates than other amino acids.

(1) Calculating the percentage losses of histidine in the excretion of urine, faeces and sweat

The % compositions of the amino acids in sweat derived from those reported in the literature (Dunstan et al 2015). The values for the urine were derived from the data in Table 4 and summarised in Table A1. The values for faeces were not available and the general body composition of amino acids from key body proteins was used as presented in Table A1.

- The excretion losses were partitioned into 3 components *(Tessari, 2006)*

    - 202 mg protein/Kg BW/day from urine,
    - 41 mg protein/Kg BW/day from faeces,
    - 129 mg protein/Kg BW/day from sweat…. >>> generating a total of 372g protein equivalent ONL /Kg BW/ day
- The average % histidine in horse urine was 1.9% (rounded in Table A1)
    - The histidine output in urine was thus 0.019 x 202g = 3.838mg / Kg BW
- The % histidine in horse faeces was 3.2% (rounded in Table A1)
    - The histidine output in faeces was thus 0.03238 x 41g = 1.327mg / Kg BW
- The % histidine in sweat was partitioned into 3 components:
    - The % free histidine in horse sweat was 6.411% (rounded to 6.4% in Table A1)
        - The histidine output in sweat was thus 0.06411 x 5g = 0.32mg / Kg BW
    - The % histidine in horse sweat proteins was 0.470% (rounded to 0.5% in Table A1)
        - The histidine output in sweat proteins was thus 0.0047 x 78g = 0.37mg / Kg BW
    - The % histidine in horse sweat skin proteins was 3.328% (rounded to 3.2% in Table A1)
        - The histidine output in sweat skin proteins was thus 0.03328 x 46.26g = 1.54mg / Kg BW
- The final % composition of the excreted amino acids was then calculated as the sum of these contributions divided by the total excreted
    - =(3.838 + 1.327 + 0.32 + 0.37 + 1.54)/372 = 7.395/372 = 0.0199 or 2.0% % as shown in Table A1
        - Ie $\%AA_{Horse\ exc}$ = 2.0%

(H) Calculating the average histidine losses in urine, faeces and sweat to derive an adjusted nitrogen balance for the 500Kg horse.

The proportion of histidine excreted per day from the ingested protein was thus 0.3007x 0.0199 x 186 = 1.11g

The proportion of histidine excreted per day from the endogenous protein turnover was 0.6999 x 0.0.0199 x 186 = 2.6023g

Combined, these represent a usage of <u>– 3.7123g</u> (rounded to -3.7 in (G) Table 5)

This was used to calculate an adjusted nitrogen balance, H = A + B + C + D + G = 2.5127 g histidine /day for a 500Kg horse (entered as 2.5g in Table 5).
