# Supplementary material for: Modelling of amino acid turnover in the horse during training and racing: A basis for developing a novel supplementation strategy
Source: PLoS One. 2020 Jan 3;15(1):e0226988. doi: 10.1371/journal.pone.0226988 (PMC6941815; doi:10.1371/journal.pone.0226988)
Supplement: Attachment — Submitted filename: Response to Reviewers 18112019.docx [file pone.0226988.s006.docx]

Journal Requirements:

1. When submitting your revision, we need you to address these additional requirements.
Please ensure that your manuscript meets PLOS ONE's style requirements, including those for file naming. The PLOS ONE style templates can be found at
[http://www.journals.plos.org/plosone/s/file?id=wjVg/PLOSOne_formatting_sample_main_body.pdf](https://protect-au.mimecast.com/s/3lRvCWLV1GIXGP2tKjdty?domain=journals.plos.org) and [http://www.journals.plos.org/plosone/s/file?id=ba62/PLOSOne_formatting_sample_title_authors_affiliations.pdf](https://protect-au.mimecast.com/s/7pLlCXLWPJID0q1hkBDPH?domain=journals.plos.org)

The title page and main body headings have been adjusted as requested.

2.  Please include captions for your Supporting Information files at the end of your manuscript, and update any in-text citations to match accordingly. Please see our Supporting Information guidelines for more information: [http://journals.plos.org/plosone/s/supporting-information](https://protect-au.mimecast.com/s/em-bCYW8ZKUpONQCMuaeK?domain=journals.plos.org).

The supplementary data have been provided as separate files with filenames preceding with S as requested. The information captions within the manuscript have been adjusted to the suggested format eg S1_Table.docx

3. Thank you for stating the following in the Acknowledgments Section of your manuscript:

Horsepower Pty Ltd represent an Australian industry partner with an interest in developing effective supplements in the equine industry.
This company had no role in study design, data collection and analysis, decision to publish, or preparation of the manuscript.
Dr David Wood works as a veterinarian consultant to Horsepower to provide independent advice. Dr Wood assisted us in the final stages of preparation of the manuscript and provided valuable insight for checking the protein model and clinical interpretation of the data.474 The university authors have not been paid as consultants and have not received any direct grant funding from Horsepower Pty Ltd.

We note that you have provided funding information that is not currently declared in your Funding Statement. However, funding information should not appear in the Acknowledgments section or other areas of your manuscript. We will only publish funding information present in the Funding Statement section of the online submission form.

Please remove any funding-related text from the manuscript and let us know how you would like to update your Funding Statement. Currently, your Funding Statement reads as follows:

Funding has been removed from the manuscript.

The conflicts of interest statement has been amended as requested in the responding letter to the editor. It did not appear possible to update this during the submission process.

The welfare of animals statement has been removed from the manuscript and supplied in the submission process.

No - The funders had no role in study design, data collection and analysis, decision to publish, or preparation of the manuscript.

Additionally, because some of your funding information pertains to [commercial funding//patents], we ask you to provide an updated Competing Interests statement, declaring all sources of commercial funding.

In your Competing Interests statement, please confirm that your commercial funding does not alter your adherence to PLOS ONE Editorial policies and criteria by including the following statement: "This does not alter our adherence to PLOS ONE policies on sharing data and materials.” as detailed online in our guide for authors  [http://journals.plos.org/plosone/s/competing-interests](https://protect-au.mimecast.com/s/1XoiCZY1gLS82DECPplcj?domain=journals.plos.org).  If this statement is not true and your adherence to PLOS policies on sharing data and materials is altered, please explain how.

This has been updated as requested.

Please include the updated Competing Interests Statement and Funding Statement in your cover letter. We will change the online submission form on your behalf.

This has been done – I could not change this in the submission process.

Please know it is PLOS ONE policy for corresponding authors to declare, on behalf of all authors, all potential competing interests for the purposes of transparency. PLOS defines a competing interest as anything that interferes with, or could reasonably be perceived as interfering with, the full and objective presentation, peer review, editorial decision-making, or publication of research or non-research articles submitted to one of the journals. Competing interests can be financial or non-financial, professional, or personal. Competing interests can arise in relationship to an organization or another person. Please follow this link to our website for more details on competing interests: [http://journals.plos.org/plosone/s/competing-interests](https://protect-au.mimecast.com/s/1XoiCZY1gLS82DECPplcj?domain=journals.plos.org)

Reviewers' comments:

Reviewer's Responses to Questions

**Comments to the Author**

1. Is the manuscript technically sound, and do the data support the conclusions?

The manuscript must describe a technically sound piece of scientific research with data that supports the conclusions. Experiments must have been conducted rigorously, with appropriate controls, replication, and sample sizes. The conclusions must be drawn appropriately based on the data presented.

Reviewer #1: Yes

Reviewer #2: Partly

2. Has the statistical analysis been performed appropriately and rigorously?

Reviewer #1: Yes

Reviewer #2: Yes

3. Have the authors made all data underlying the findings in their manuscript fully available?

The [PLOS Data policy](https://protect-au.mimecast.com/s/7-2jC1WLMyUB5k1TywJrk?domain=plosone.org) requires authors to make all data underlying the findings described in their manuscript fully available without restriction, with rare exception (please refer to the Data Availability Statement in the manuscript PDF file). The data should be provided as part of the manuscript or its supporting information, or deposited to a public repository. For example, in addition to summary statistics, the data points behind means, medians and variance measures should be available. If there are restrictions on publicly sharing data—e.g. participant privacy or use of data from a third party—those must be specified.

Reviewer #1: Yes

Reviewer #2: Yes

4. Is the manuscript presented in an intelligible fashion and written in standard English?

PLOS ONE does not copyedit accepted manuscripts, so the language in submitted articles must be clear, correct, and unambiguous. Any typographical or grammatical errors should be corrected at revision, so please note any specific errors here.

Reviewer #1: Yes

Reviewer #2: Yes

5. Review Comments to the Author

Please use the space provided to explain your answers to the questions above. You may also include additional comments for the author, including concerns about dual publication, research ethics, or publication ethics. (Please upload your review as an attachment if it exceeds 20,000 characters)

Reviewer #1: In this work the authors' purpose was to analyse published rates of protein intake, turnover and excretion and extrapolated these to individual appraisals of amino acids in sports horses.

The work turns out to be well written, the references cited are appropriate. The division into four categories is a wright decision. The choice of two types of protein is clear and well described. The results are well described. The methods are well described.

The results are very interesting. Indeed, the results will lay important bases on understanding biochemistry of physical exercise and physiological interactions on protein turnover homeostasis. Not only that, they will also allow to better understand the type of supplement to be carried out during a training and if it is the case to carry it out.

Major revision:
1) The format result and discussion must to be divided.

The manuscript body formatting guidelines state that this section should appear as “Results and discussion” and this is how the manuscript was developed. The nature of the project and development of concepts leans itself very strongly to this style of format.

[http://www.journals.plos.org/plosone/s/file?id=wjVg/PLOSOne_formatting_sample_main_body.pdf](https://protect-au.mimecast.com/s/3lRvCWLV1GIXGP2tKjdty?domain=journals.plos.org)

Comments:
a) Could be a possibility to compare these results on possible horses supplement strategy and humans supplement?

This is an excellent suggestion. The results from the human modelling exercise (see reference 1) concluded that supplementation in the order of 5 grams of amino acids a day representing just 6% of the total protein intake for a 70Kg individual, could provide benefit if taken immediately or after exercise. The horse modelling concluded that 20-80g per day for a 500Kg animal, representing 2.3 - 9.3% of the total protein intake, could provide benefit in maintaining a positive nitrogen balance and thus reduce demand on muscle turnover for supply.

This comparison has been added to the conclusions.

Reference 1:

*Dunstan, R.H.; Macdonald, M.M.; Murphy, G.R.; Thorn, B.; Roberts, T.K. Modelling of protein turnover provides insight for metabolic demands on those specific amino acids utilised at disproportionately faster rates than other amino acids. Amino Acids* ***2019****, 10.1007/s00726-019-02734-1, doi:10.1007/s00726-019-02734-1.*

b) Could be used The Modelling of amino acid turnover to analyse the differences into amino acid turnover in human metabolism during competition or exercise?

This could be done and will represent a future application of the modelling process. This is especially important in future work as we have demonstrated that at least three different “types” of sweating can occur in high performance athletes: one type loses lower quantities of amino acids and at the other end of the spectrum, another type loses amino acids at very high concentrations. In addition, the excretion profiles are slightly different between the types and there could be an opportunity to really tailor the modelling to meat individual demands - see reference:

*Dunstan, R. H., Sparkes, D. L., Dascombe, B. J., Macdonald, M. M., Evans, C. A., Stevens, C. J., Gottfries, J. . . Roberts, T. K. (2016). Sweat facilitated amino acid losses in male athletes during exercise at 32-34°C. PLoS ONE, 11(12). doi:*[*10.1371/journal.pone.0167844*](http://dx.doi.org/10.1371/journal.pone.0167844)

Reviewer #2: PLOS One Article Review
Modelling of amino acid turnover in the horse during training and racing: a basis for developing a novel supplementation strategy

Comments to Authors:

Little is known in regard to amino acid requirements in horses of all life stages; therefore, research investigating changes in amino acid turnover is an important step in determining amino acids of high priority and future dietary requirements. The objective of this study was to develop a theoretical model from previously published data to determine amino acid demand during exercise. While there are significant limitations associated with the development of this type of theoretical model, these data may still have a significant impact to the scientific community.

General Statements:

The manuscript is well-written and has the potential to positively impact the industry. While the information provided in the manuscript is valuable, the true balance of amino acids remains unclear because of their complex utilization in a multitude of metabolic and physiological pathways. I commend authors for accounting for, and acknowledging, certain variabilities and limitations; however, the data must be interpreted and utilized with caution. Ultimately, the equine industry is severely lacking the scope of research required to determine dietary requirements through a modeling study at this time. These data provide a nice mathematical indication of which amino acids should be of high priority in future amino acid research to accurately determine dietary requirements throughout various stages of life and performance intensities.

Major Concerns:

Authors do the best they can with the data available to develop a theoretical model; however, the results and conclusions must be interpreted with caution. The paper elucidates specific amino acids that should be of high priority in future animal studies thus the value to the industry; however, the industry remains off-pace in physiological research to determine dietary requirements through the use of a modelling system.

Line 421: While the model does a nice job of estimating positive and negative amino acid balance, the fed rations are variable (as stated in the paper) and not necessarily representative of all horses in heavy work. I caution authors against suggesting a specific supplementation rate due to the theoretical nature of the model and the limited biological research in amino acid nutrition, synthesis, turnover, metabolism, etc. in the horse. See general statements.

We have not made specific recommendations for supplementation rates, but we have proposed a novel strategy of replenishing those amino acids that were shown to be in negative nitrogen balance under certain conditions. The modelling provides a framework to show that certain amino acids would be in greater demand than others due to losses via excretion pathways. The modelling also provided a realistic approach to estimating the likely levels of key amino acids that would be required for effective supplementation. This has never been previously attempted. This at least provides a starting point for determining levels of supplementation and their compositions for future testing. As stated in the conclusion, the next phase is to look at trials under a range of scenarios, training regimes and breeds.

The final statements in the conclusions echo the cautions from the reviewer:

*“….. Applied research with horses is now required to determine whether this supplementation approach can be effective in a range of breeds and competition formats. Future research should also focus on ascertaining specific amino acid requirements at various stages of training and competition to determine whether there would be long term benefits for horses such as better maintenance of muscle mass, optimising performance and reducing muscle damage.”*

Line 440-442: I caution authors against using the verbiage “immediately after exercise”. Simply removing “immediately” from the statement would suffice.

“immediately” has been removed from the conclusions.

Tables: Be consistent in formatting of tables.

Shading has been removed from the tables.

Minor Concerns:
Line 148: Insert a space between 52% and of.

Done
Line 300: Add “of” in “various types of competition”

Done
Line 302-303: Change “initially run by assuming that…” to “initially run on the assumption that the …”

Done
Line 312: remove “the” before urine and sweat; remove “the” before supplementary data

Done
Line 313: Add comma after ornithine

I don’t think there should be a comma before a conjunction.
Line 314: Add comma before respectively

Done
Line 320: Serine is not the most abundant component of sweat, but may be the most abundant amino acid in sweat. Change “component” to “amino acid”

Done
Line 322: Remove “the” before horse sweat fluid.

Done
Line 323: Remove “the” before horse plasma

Done
Line 324: Remove “the” before horse sweat

Done

Table 5. Make sure title is above the table. It is currently split with part of the title above the table and the last line below the table.

Done

Line 326-327: “has involvement” should be “is involved”

Done
Line 331: “highly abundant” is redundant. Remove “highly”

Done
Line 333: Remove “the” before plasma

Done
Line 343: Remove “as urea”

Done
Line 344: Arginine is involved in much more than simply NO production.

This was added: “….many aspects of metabolism, including the generation of nitric oxide.”
Line 376: Remove “the” before red blood cell parameters

Done
Line 383: Remove “the” before body proteins

Done
Line 385: Remove “the” before protein turnover

Done
Line 392: Replace “has been shown to be” with “is”… Histidine is required for adequate synthesis…

Done
Line 394: Add comma after haemoglobin

Done
Line 400: Remove “supporting”

Done
Line 420: Why have glutamine separate instead of in the list of amino acids suggested for supplementation? Instead, “this model would suggest that histidine, lysine, valine, phenylalanine, and glutamine should also be supplemented”

Because Glx and serine were identified in the general modelling – the other amino acids become limiting when there is a stimulus under fitness training to build muscle and aerobic capacity.

6. PLOS authors have the option to publish the peer review history of their article ([what does this mean?](https://protect-au.mimecast.com/s/N59pC2xMX0TZORvu0P9QH?domain=journals.plos.org)). If published, this will include your full peer review and any attached files.


If you choose “no”, your identity will remain anonymous but your review may still be made public.


**Do you want your identity to be public for this peer review?** For information about this choice, including consent withdrawal, please see our [Privacy Policy](https://protect-au.mimecast.com/s/d1jEC3QN3Af90GwTOX7fM?domain=plos.org).

Reviewer #1: No

Reviewer #2: No
